# Supplementary material for: MSA: reproducible mutational signature attribution with confidence based on simulations
Source: BMC Bioinformatics. 2021 Nov 4;22:540. doi: 10.1186/s12859-021-04450-8 (PMC8567580; doi:10.1186/s12859-021-04450-8)
Supplement: Supplementary file 1 — Additional file 1: Comparison of MSA performance with existing tools. Performance of MSA benchmarked using real PCAWG consortium data and simulations, compared with SigProfiler and SignatureAnalyzer tools. [file 12859_2021_4450_MOESM1_ESM.pdf]

Comparison of MSA performance with existing tools

Performance of MSA was benchmarked against existing tools using both real and synthetic data published by the PCAWG consortium, available at the following Synapse link: https://www.synapse.org/#!Synapse:syn11726601/wiki/513478

Comparisons of signature attributions extracted from 2,780 PCAWG cancer genomes are shown on Figure 1 using various similarities between the original and reconstructed mutational spectra. The performance appears comparable across the tools, however, since the ground truth is not known, it is difficult to determine which tool performs best on real data.

Benchmarking can be more informative when using synthetic data where the ground truth is known. At first, the performance was tested using simulations mimicking real PCAWG data, available at https://www.synapse.org/#!Synapse:syn18500213. These simulations include 2700 synthetic whole-genome mutational spectra, with 300 spectra from each of 9 cancer types of the PCAWG dataset, for single base substitutions in the 96 trinucleotide context generated from COSMIC reference signatures.

The performance was also tested on simulations of randomly generated signatures and exposures, available at https://www.synapse.org/#!Synapse:syn18500221.

Along with the simulated spectra and the ground truth signature activities, PCAWG consortium shared the results of signature extraction and attribution for SigProfilerExtract and SignatureAnalyzer tools, which are compared to MSA ran on all available or SigProfiler-extracted signatures.

For simulations mimicking real PCAWG data and based on COSMIC signatures, Figure 2 (a) shows the benchmarking results using various metrics, including sensitivity, specificity, precision, accuracy, F1 and MCC (Matthews Correlation coefficient). Figure 2 (b) shows the similarities of the reconstructed mutation spectra with the ground truth spectra using cosine similarity and other metrics. Figures 4 and 5 show similar metrics for each simulated signature.

Similarly, Figures 3 as well as Figures 6-8 show benchmarking results derived from simulations of randomly generated signatures and exposures.

It has to be noted that such comparison is only aimed at the evaluation of signature attribution performance, rather signature extraction. Both SigProfiler and SignatureAnalyzer are extracting signatures de-novo, and then decomposing or matching them to the reference signatures. On the other hand, MSA only performs signature attribution with a given signature catalogue, either a whole COSMIC reference catalogue (shown as MSA_allsigs in the plots), or a catalogue given by SigProfiler decomposition, shown as MSA_SP. When randomly generated signatures are used, MSA_allsigs label refers to results obtained with the catalogue of all 30 randomly generated signatures.

In the case of simulations based on COSMIC signatures, it can be seen that the best performance is achieved with MSA attribution based on the signatures taken from the SigProfiler decomposition – where MSA is run on the SigProfiler output due to the native integration of the tool. However, MSA attribution based on the whole COSMIC catalogue shows a comparable performance, due to the fact that the simulations were made only with the signatures taken from the reference catalogue. Therefore, the recommended way to run MSA is using the SigProfiler output, as

it makes sure that any novel signatures that may be present in the cohort are accurately attributed.

In the case of simulations based on randomly generated signatures, MSA shows best performance when the whole catalogue of randomly generated signatures is used (MSA_allsigs label). However, as the whole set of reference signatures is generally not known, the real-life scenario corresponds to MSA attributions based on de-novo extracted signatures (MSA_SP label).

The reason why MSA attribution generally achieves higher performance metrics than that of other tools is due to its automised optimisation of the penalties applied, coupled with application of confidence intervals. SigProfiler uses fixed penalties of 0.01/0.05, which appear to be too conservative in many cases, with the exception of scenarios with randomly generated Poisson signatures that are difficult to discern from noise and each other. SignatureAnalyzer, on the other hand, tends to overfit the data as activities of virtually all signatures are attributed in each sample, i.e. no regularisation appears to be implemented. Expectedly, this leads to a very high sensitivity, but also low specificity.

Similarity metrics were calculated from normalised reconsturcted and original mutation spectra, defined as follows:

- Cosine similarity: $\frac{x \cdot y}{||x||_2 \cdot ||y||_2}$
- Correlation: $\frac{(x-\overline{x}) \cdot (y-\overline{y})}{||x-\overline{x}||_2 \cdot ||y-\overline{y}||_2}$
- Manhattan (L1): $1 - ||x - y||$
- Euclidean (L2): $1 - ||x - y||_2$
- Chebyshev (L∞): $1 - \lim_{p \to \infty} ||x - y||_p = 1 - \max_i |x_i - y_i|$
- Jensen-Shannon: $1 - \sqrt{\frac{D(x||m) + D(y||m)}{2}}$, where $m = \frac{1}{2}(x + y)$ and Kullback-Leibler divergence (aka relative entropy) for two probabilities: $D(x||y) = \sum x \log \frac{x}{y}$

Performance metrics were calculated from the numbers of true positives (TP), false positives (FP), true negatives (TN) and false negatives (FN), are defined as follows:

- Sensitivity: $\frac{\mathrm{TP}}{\mathrm{TP+FN}}$
- Specificity: $\frac{\mathrm{TN}}{\mathrm{TN+FP}}$
- Precision: $\frac{\mathrm{TP}}{\mathrm{TP+FP}}$
- Accuracy: $\frac{\mathrm{TP+TN}}{\mathrm{TP+TN+FP+FN}}$
- F1: $\frac{\mathrm{2TP}}{\mathrm{2TP+FP+FN}}$
- MCC (Matthews Correlation coefficient): $\frac{\mathrm{TP*TN-FP*FN}}{\sqrt{\mathrm{(TP+FP)(TP+FN)(TN+FP)(TN+FN)}}}$

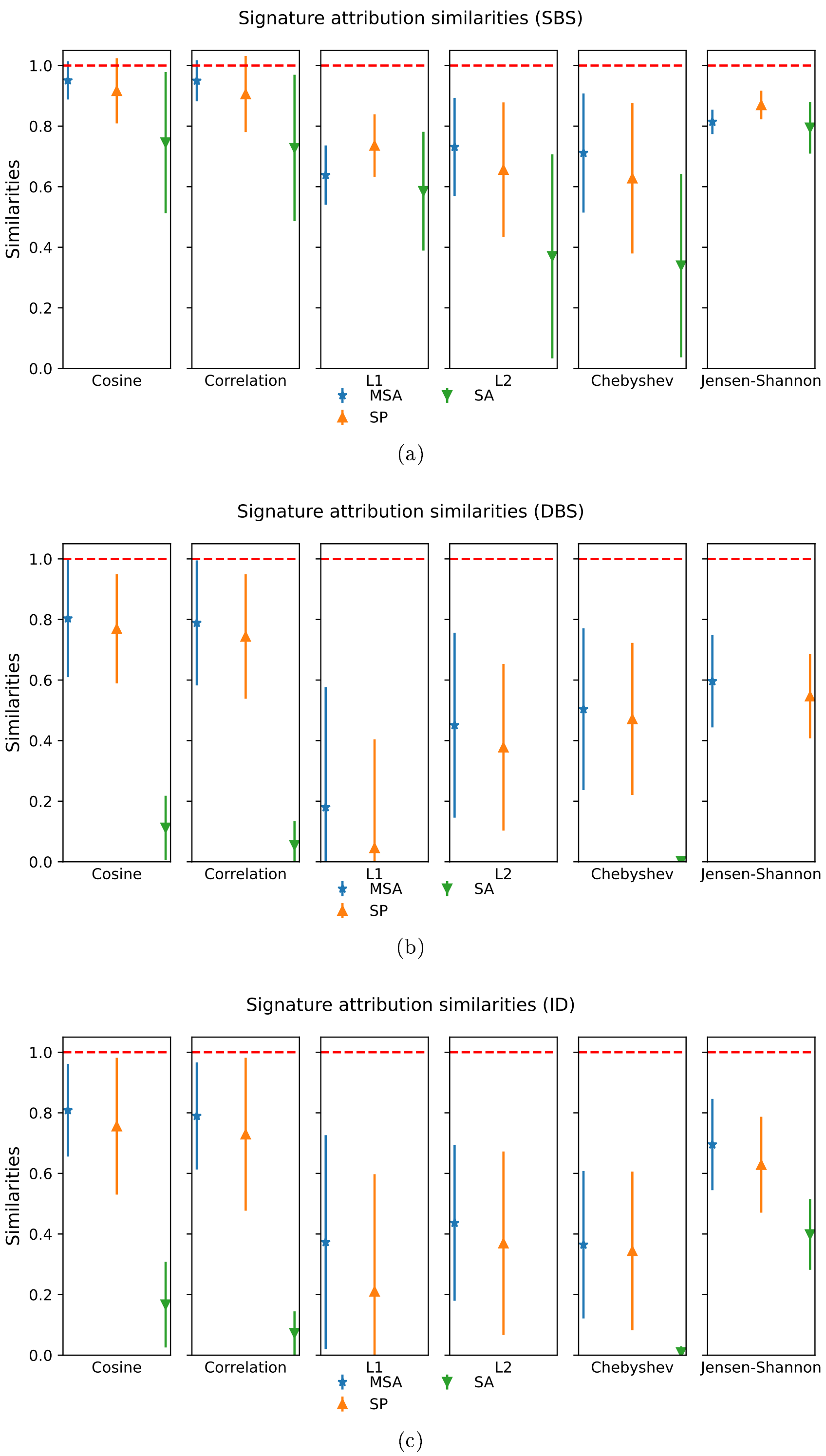

Figure 1 Similarities between the original and reconstructed spectra for real PCAWG data, measured using cosine similarity, correlation, L1 and L2 similarities, Chebyshev and Jensen-Shannon similarities. (a) Single base substitutions in SBS-96 classification, (b) Doublet base substitutions in DBS-78 classification, (c) Indels in ID-83 classification. MSA is compared to SigProfiler (SP) and SignatureAnalyzer (SA) tools. Markers represent the means of distributions, whereas error bars represent standard deviations.

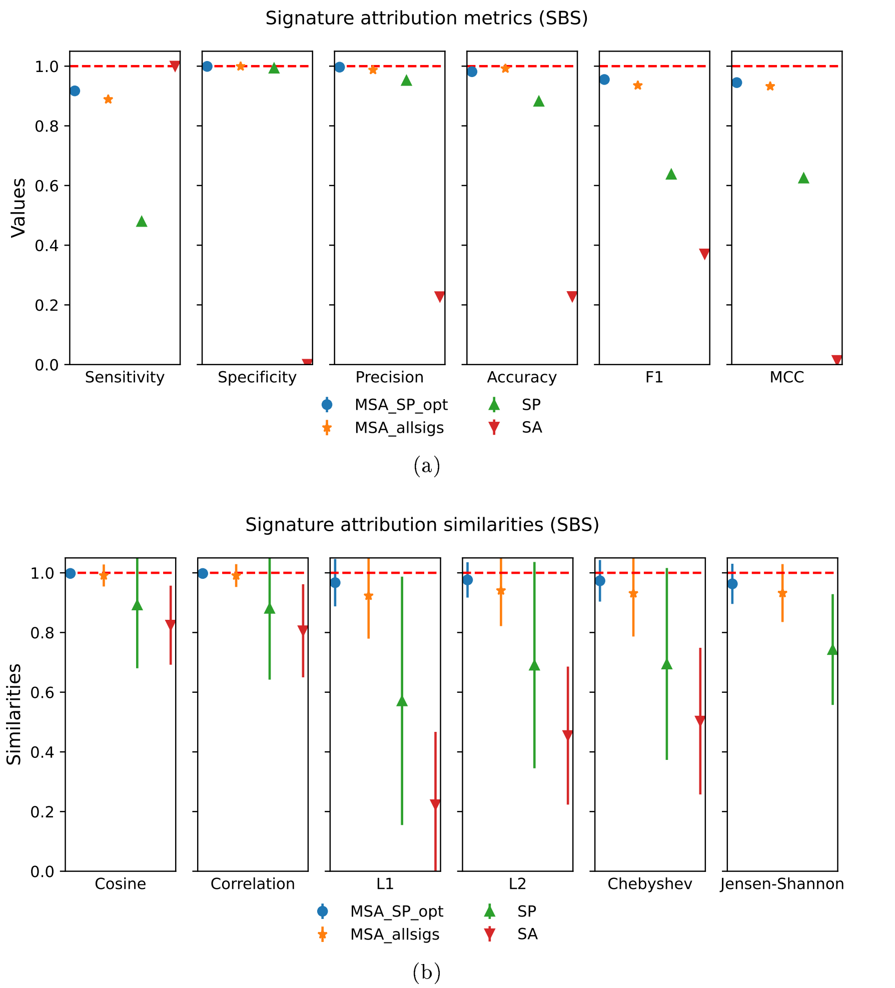

Figure 2 Simulations based on COSMIC signatures. (a) Comparison of sensitivity, specificity, precision, accuracy, F1 and MCC (Matthews Correlation coefficient) for MSA, SigProfilerExtractor and SignatureAnalyzer. MSA_SP refers to MSA attribution based on SigProfiler-decomposed reference signatures, whereas MSA_allsigs refers to the MSA attribution using the whole COSMIC catalogue. (b) Similarities of reconstructed mutation spectra with the ground truth, measured using cosine similarity, correlation, L1 and L2 similarities, Chebyshev and Jensen-Shannon similarities. Markers represent the means of distributions, whereas error bars represent standard deviations.

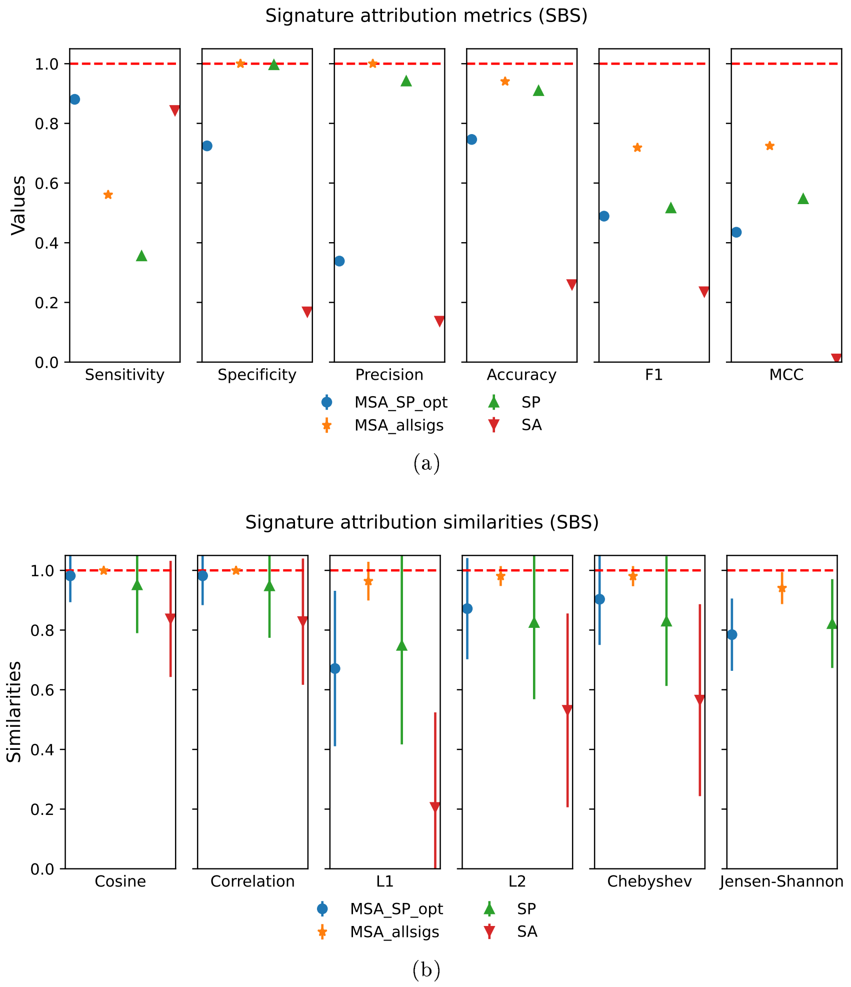

Figure 3 Simulations based on randomly generated signatures. (a) Comparison of sensitivity, specificity, precision, accuracy, F1 and MCC (Matthews Correlation coefficient) for MSA, SigProfilerExtractor and SignatureAnalyzer. MSA_SP refers to MSA attribution based on SigProfiler-decomposed reference signatures, whereas MSA_allsigs refers to the MSA attribution using the whole COSMIC catalogue. (b) Similarities of reconstructed mutation spectra with the ground truth, measured using cosine similarity, correlation, L1 and L2 similarities, Chebyshev and Jensen-Shannon similarities. Markers represent the means of distributions, whereas error bars represent standard deviations.

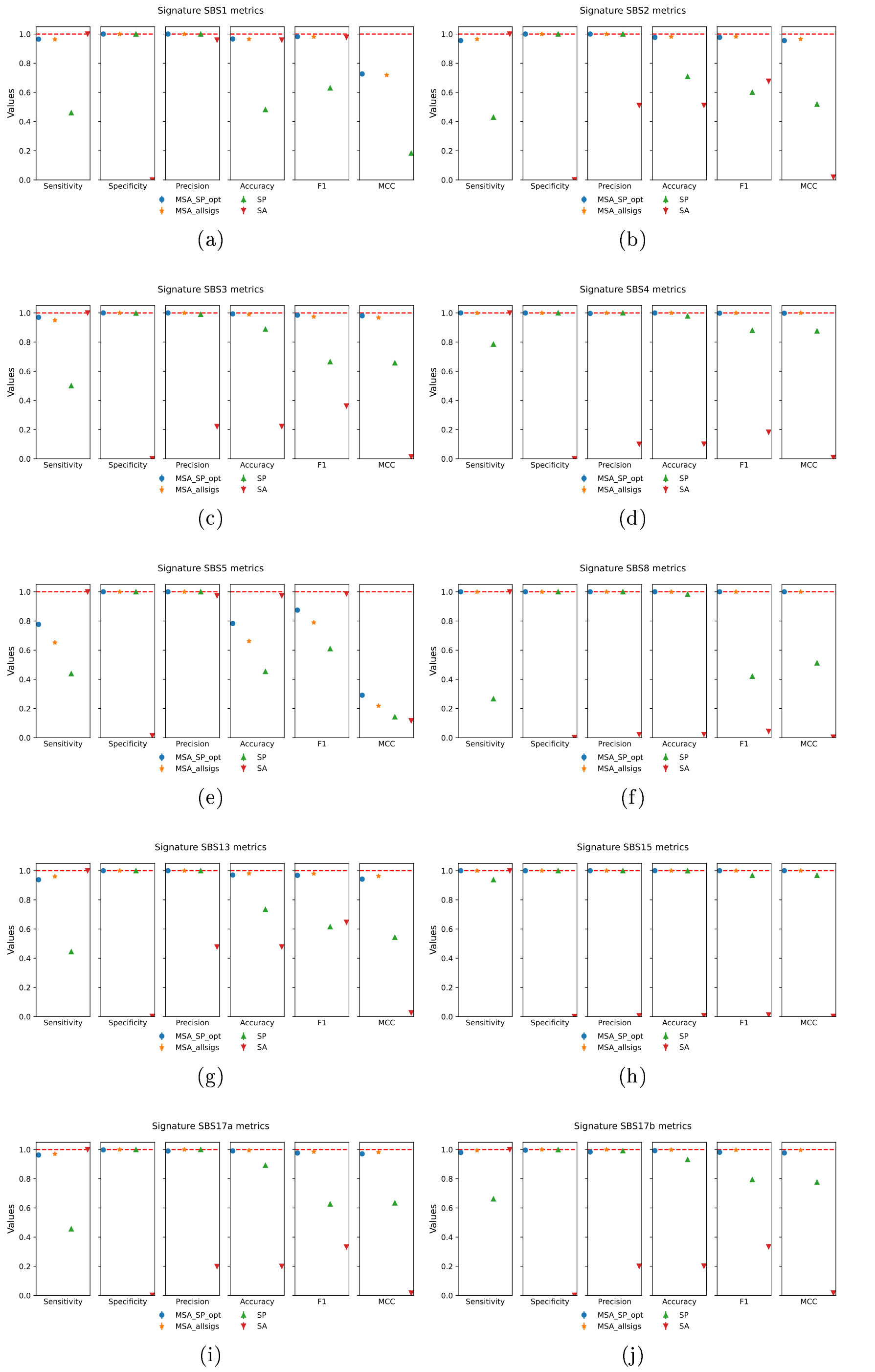

Figure 4 Simulations based on COSMIC signatures. (a-j) Signature-based comparison of sensitivity, specificity, precision, accuracy, F1 and MCC (Matthews Correlation coefficient) for MSA, SigProfilerExtractor and SignatureAnalyzer.

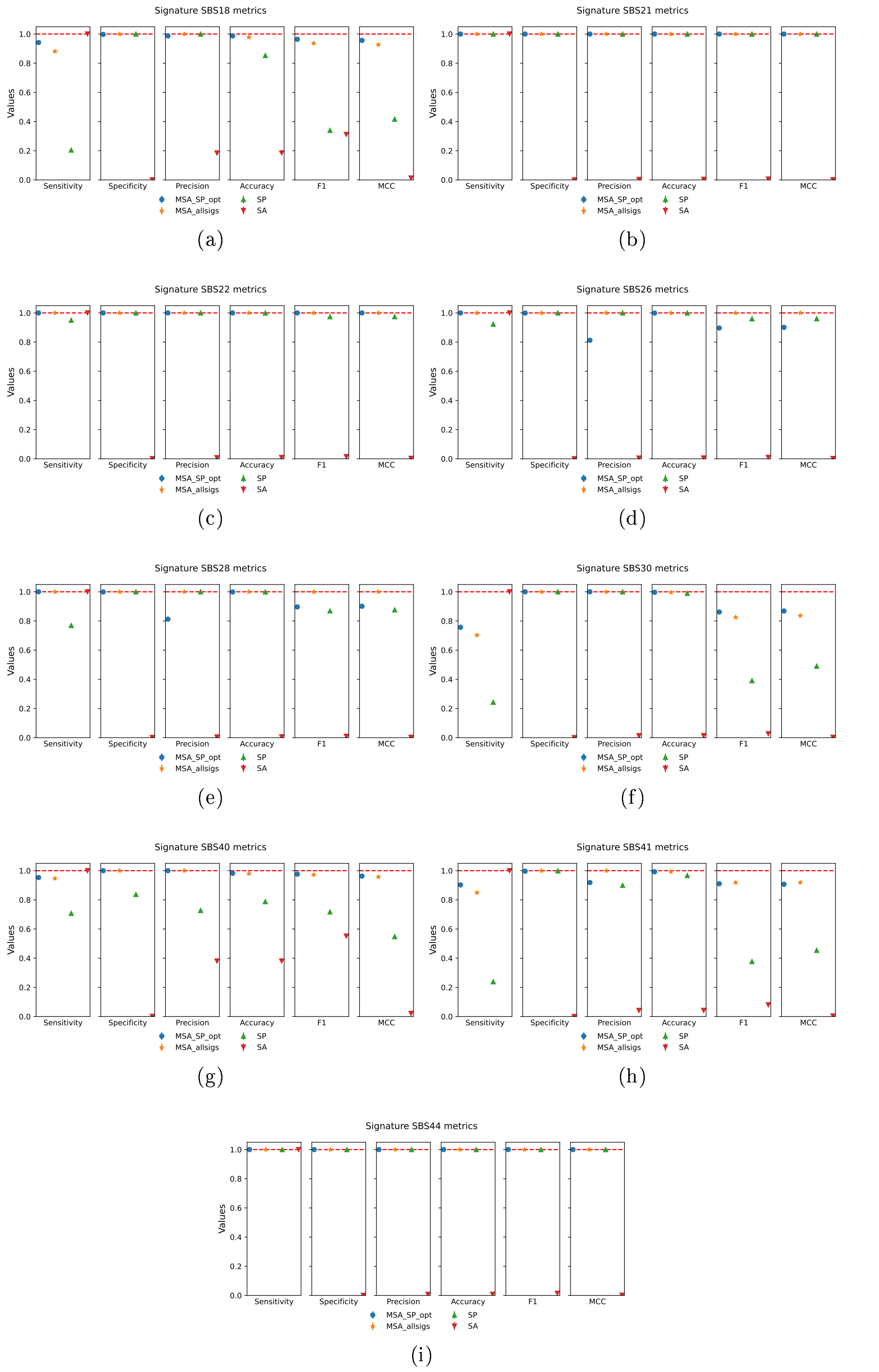

Figure 5 Simulations based on COSMIC signatures. (a-i) Signature-based comparison of sensitivity, specificity, precision, accuracy, F1 and MCC (Matthews Correlation coefficient) for MSA, SigProfilerExtractor and SignatureAnalyzer.

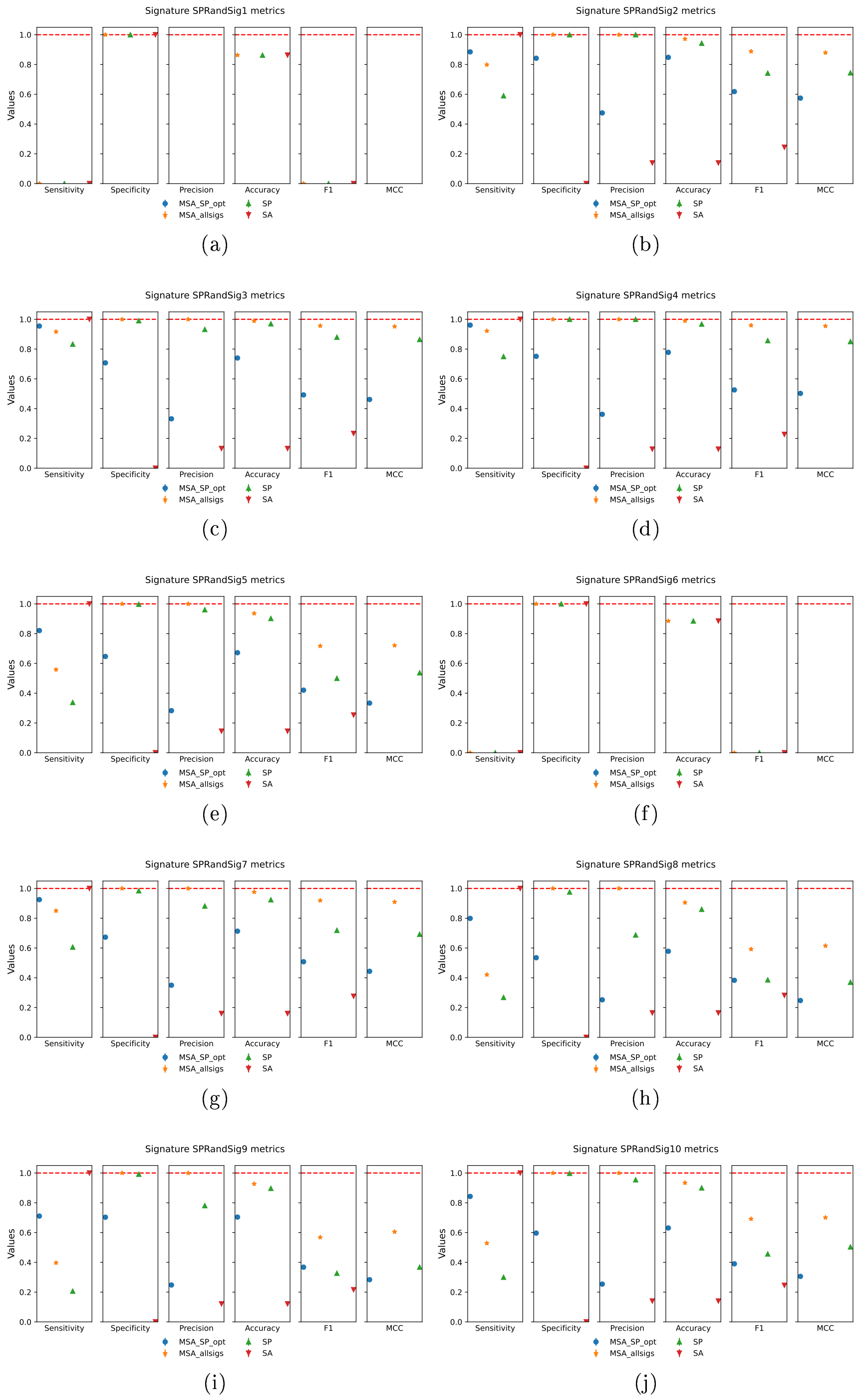

Figure 6 Simulations based on randomly generated signatures. (a-j) Signature-based comparison of sensitivity, specificity, precision, accuracy, F1 and MCC (Matthews Correlation coefficient) for MSA, SigProfilerExtractor and SignatureAnalyzer.

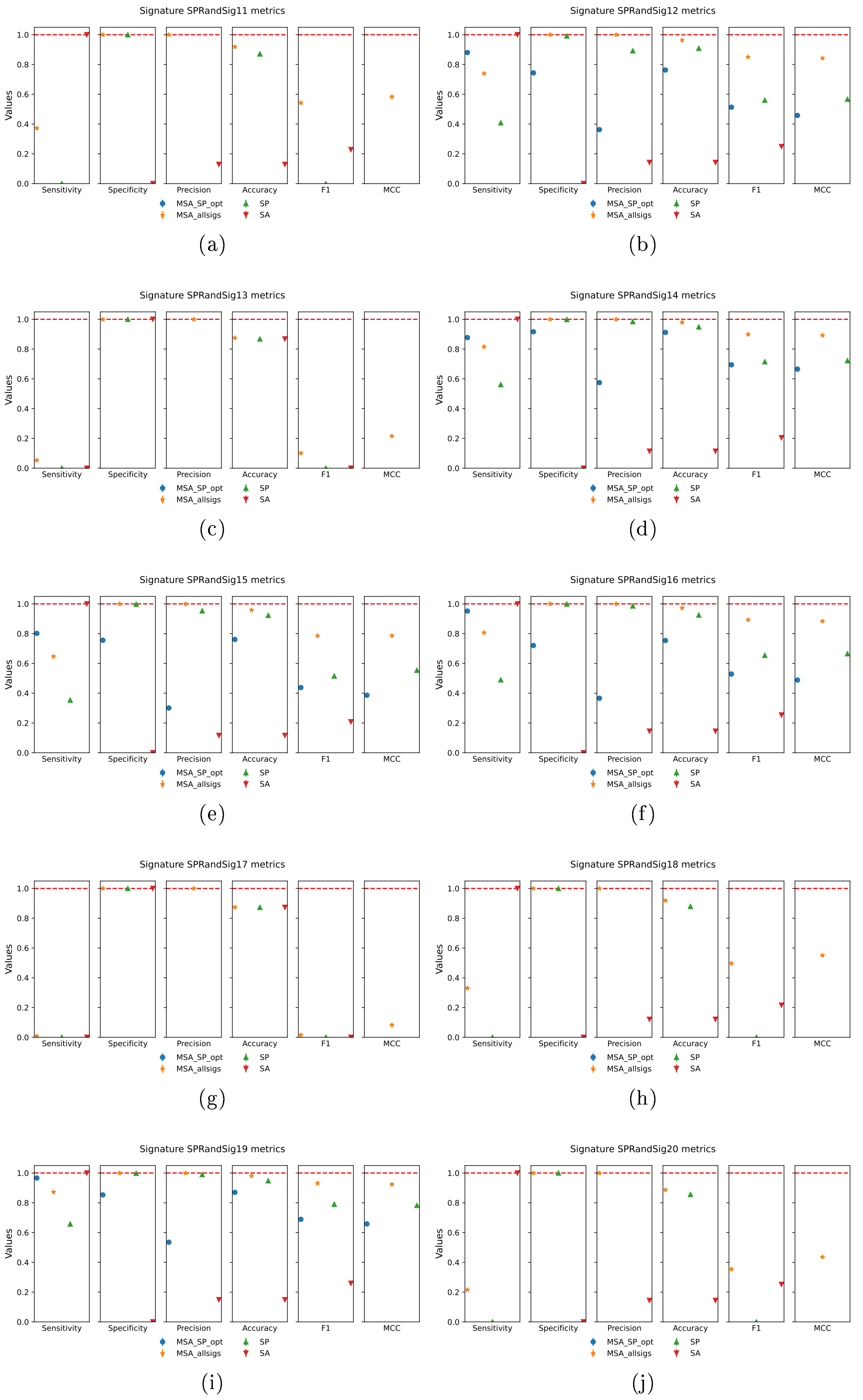

Figure 7 **Simulations based on randomly generated signatures. (a-j) Signature-based comparison of sensitivity, specificity, precision, accuracy, F1 and MCC (Matthews Correlation coefficient) for MSA, SigProfilerExtractor and SignatureAnalyzer.**

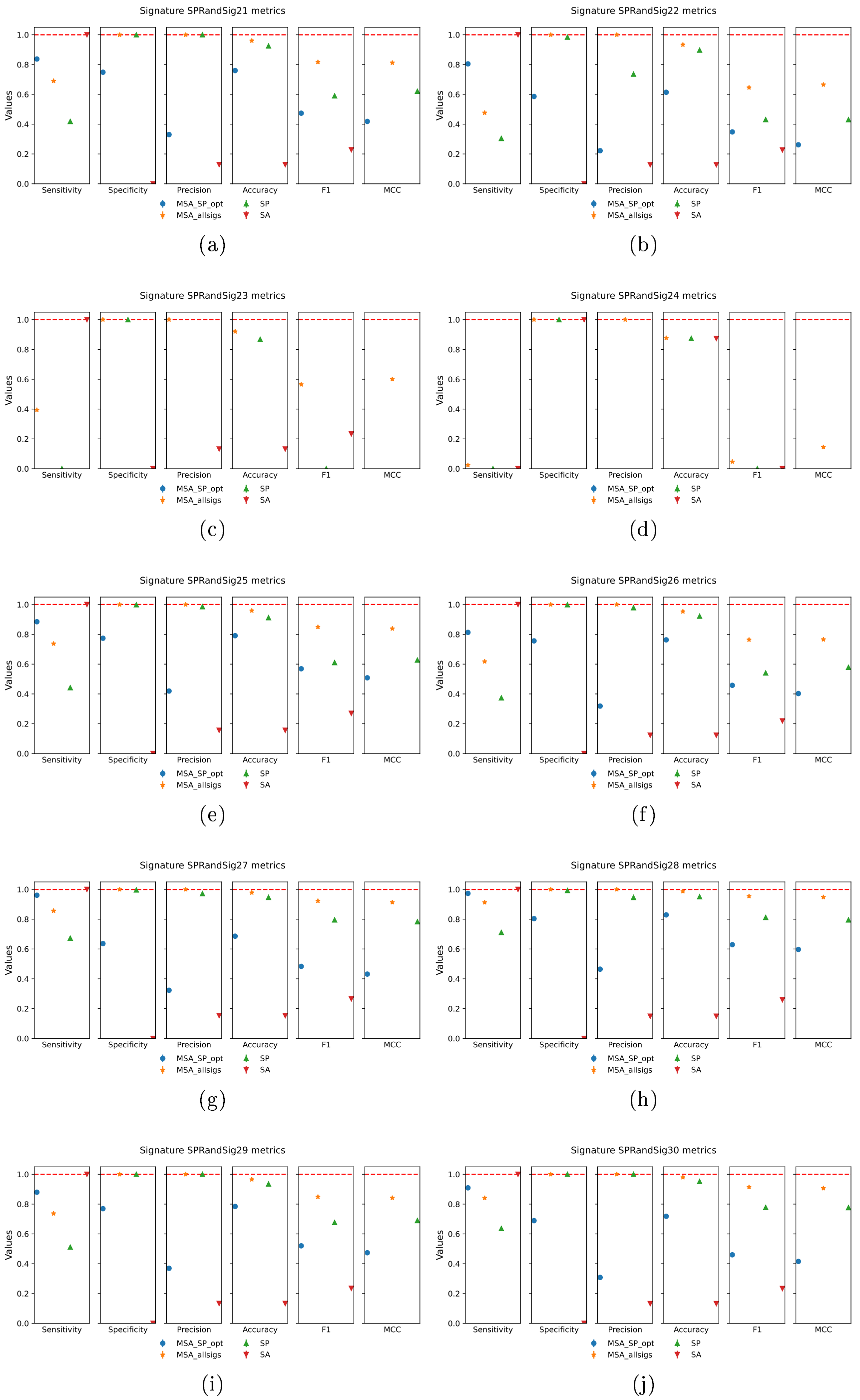

Figure 8 Simulations based on randomly generated signatures. (a-j) Signature-based comparison of sensitivity, specificity, precision, accuracy, F1 and MCC (Matthews Correlation coefficient) for MSA, SigProfilerExtractor and SignatureAnalyzer.
