# Supplementary material for: MSA: reproducible mutational signature attribution with confidence based on simulations
Source: BMC Bioinformatics. 2021 Nov 4;22:540. doi: 10.1186/s12859-021-04450-8 (PMC8567580; doi:10.1186/s12859-021-04450-8)
Supplement: Supplementary file 2 — Additional file 2: MSA performance across different mutation types on data-driven simulations. Performance of MSA validated for SBS, DBS and ID mutation types using data-driven simulations derived from the PCAWG data. [file 12859_2021_4450_MOESM2_ESM.pdf]

## MSA performance across different mutation types on data-driven simulations

Performance of MSA is measured for SBS, DBS and ID (indels) mutation types using data-driven simulations derived from the PCAWG dataset. Here, 10000 simulations were generated by running an intentionally overfitted NNLS attribution with 0 penalty on the whole PCAWG dataset (2780 samples). Resulting attributions were reshuffled with replacement and injected with Gaussian noise, with the standard deviation amounting to 10% of the mutational burden for any given sample. This approach is automised within the MSA automatic simulations routine in order to derive optimal penalties for any given input cohort.

Figure 1 shows the various metrics, including sensitivity, specificity, precision, accuracy and MCC (Matthews Correlation coefficient) across a range of penalties for (a) SBS, (b) DBS and (c) ID mutation types. Figures 2-12 show these metrics for all signatures separately.

Using the default optimisation strategy which prioritises specificity of all signatures, the optimal penalty for SBS mutation type is selected to be 0.001 L2 penalty – the value at which all signatures reach 95% specificity. Optimal penalties for DBS and ID mutation types are selected to be null, since even without optimisation, application of confidence intervals leads to a specificity of above 95% for all DBS and ID signatures. This is due to the fact that the reference signatures are well separated, hence very little or no regularisation is required in order to attribute reference signatures accurately, according to simulations.

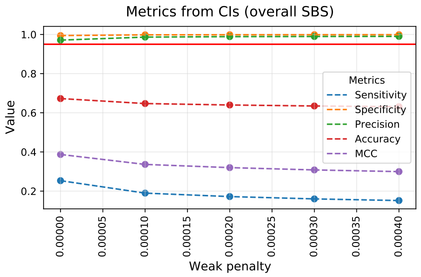


(a)

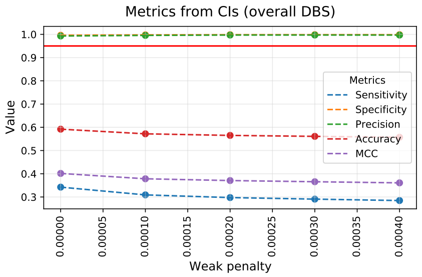


(b)

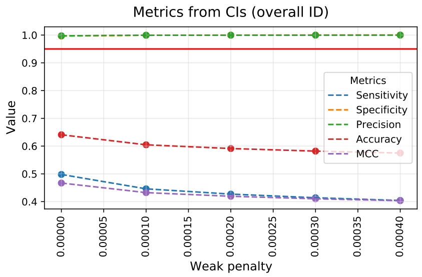


(c)

**Figure 1** MSA performance metrics: sensitivity, specificity, precision, accuracy and MCC (Matthews Correlation coefficient) for (a) SBS, (b) DBS and (c) ID mutation types, measured across all signatures.

Metrics calculated from the numbers of true positives (TP), false positives (FP), true negatives (TN) and false negatives (FN), are defined as follows:

- Sensitivity: $\frac{TP}{TP+FN}$
- Specificity: $\frac{TN}{TN+FP}$
- Precision: $\frac{TP}{TP+FP}$
- Accuracy: $\frac{TP+TN}{TP+TN+FP+FN}$
- F1: $\frac{2TP}{2TP+FP+FN}$
- MCC (Matthews Correlation coefficient): $\frac{TP*TN-FP*FN}{\sqrt{(TP+FP)(TP+FN)(TN+FP)(TN+FN)}}$

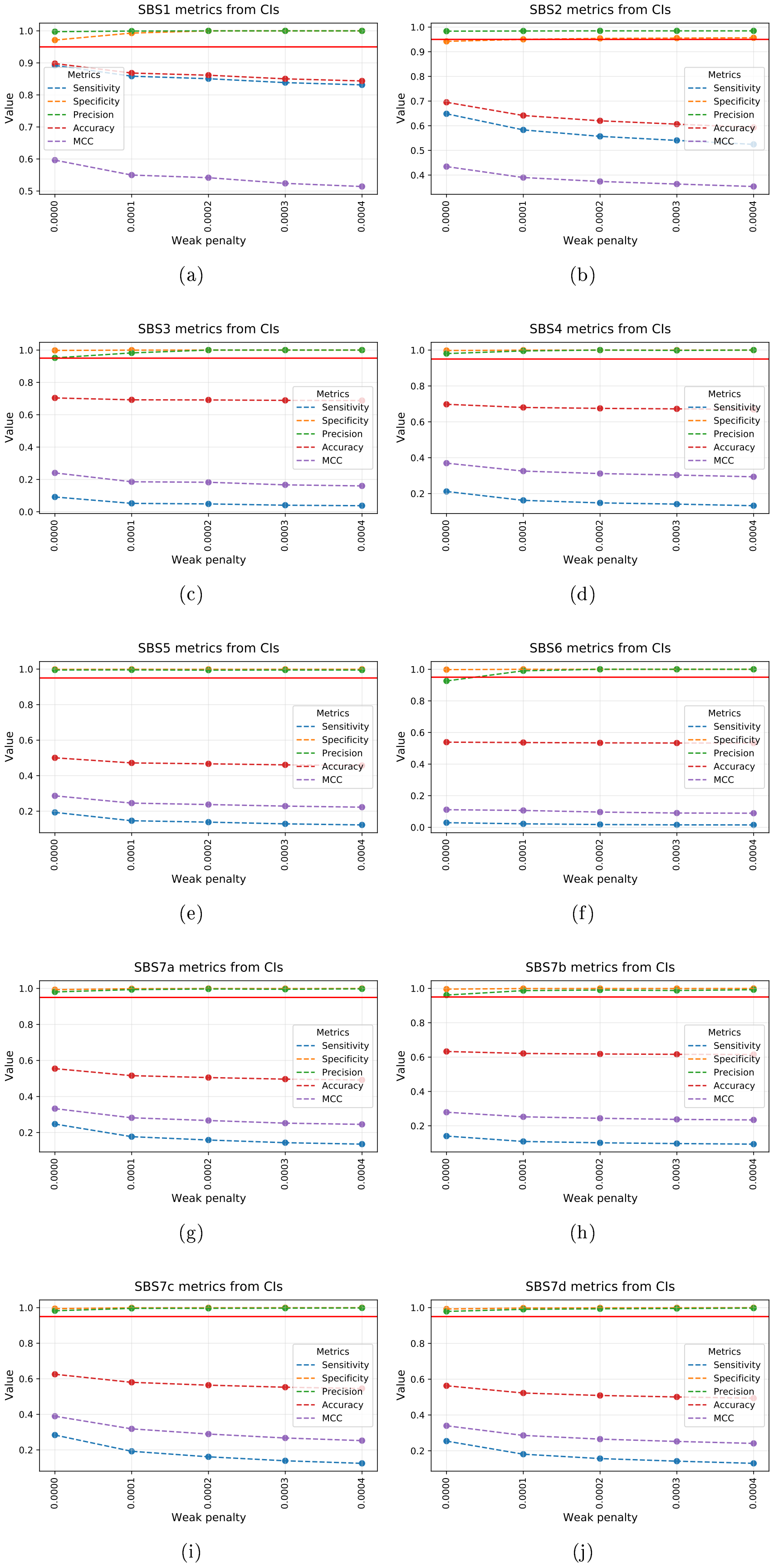


**Figure 2** MSA performance metrics: sensitivity, specificity, precision, accuracy and MCC (Matthews Correlation coefficient) measured for SBS signatures 1-7d.

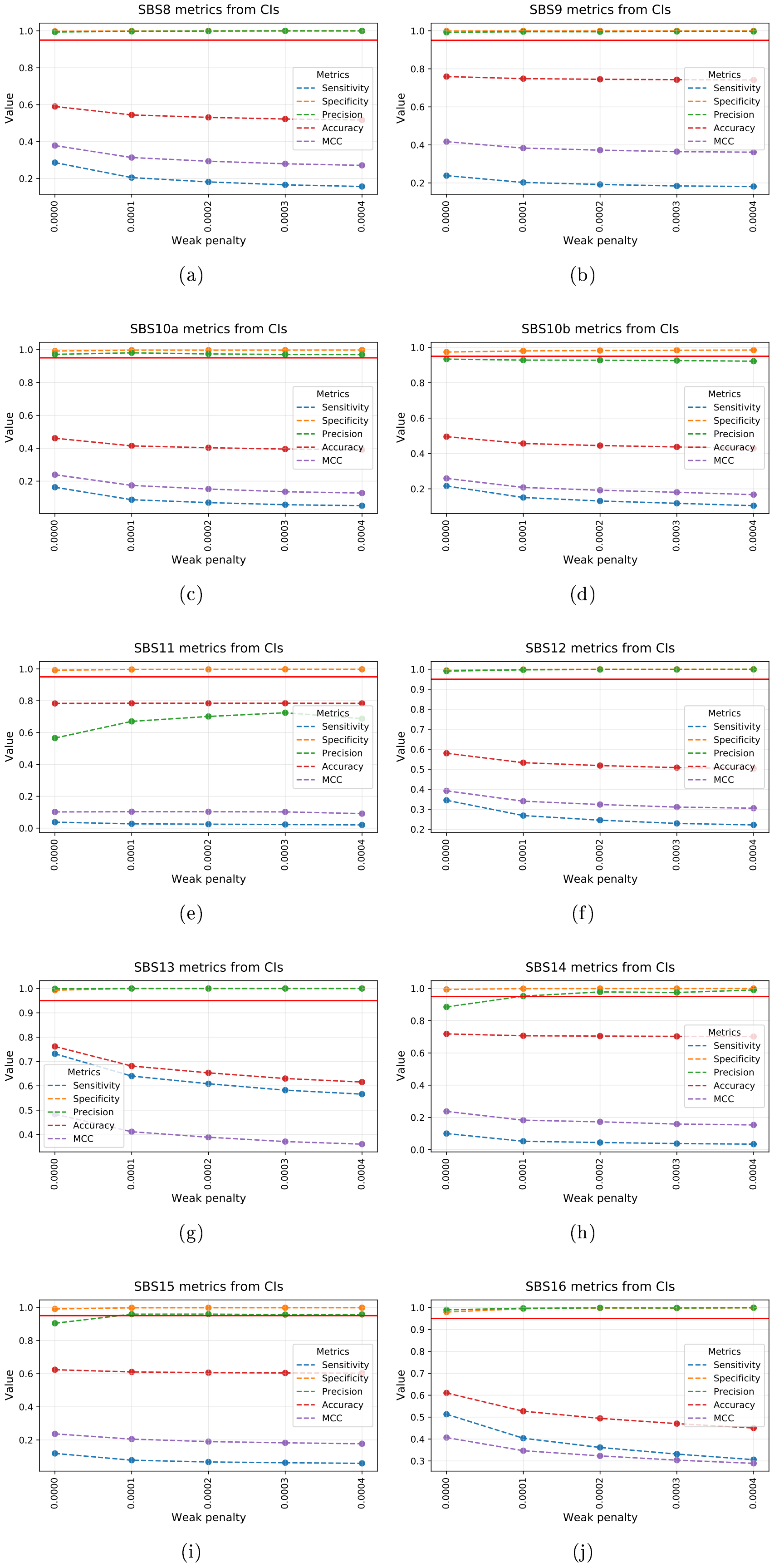


**Figure 3 MSA performance metrics: sensitivity, specificity, precision, accuracy and MCC (Matthews Correlation coefficient) measured for SBS signatures 8-16.**

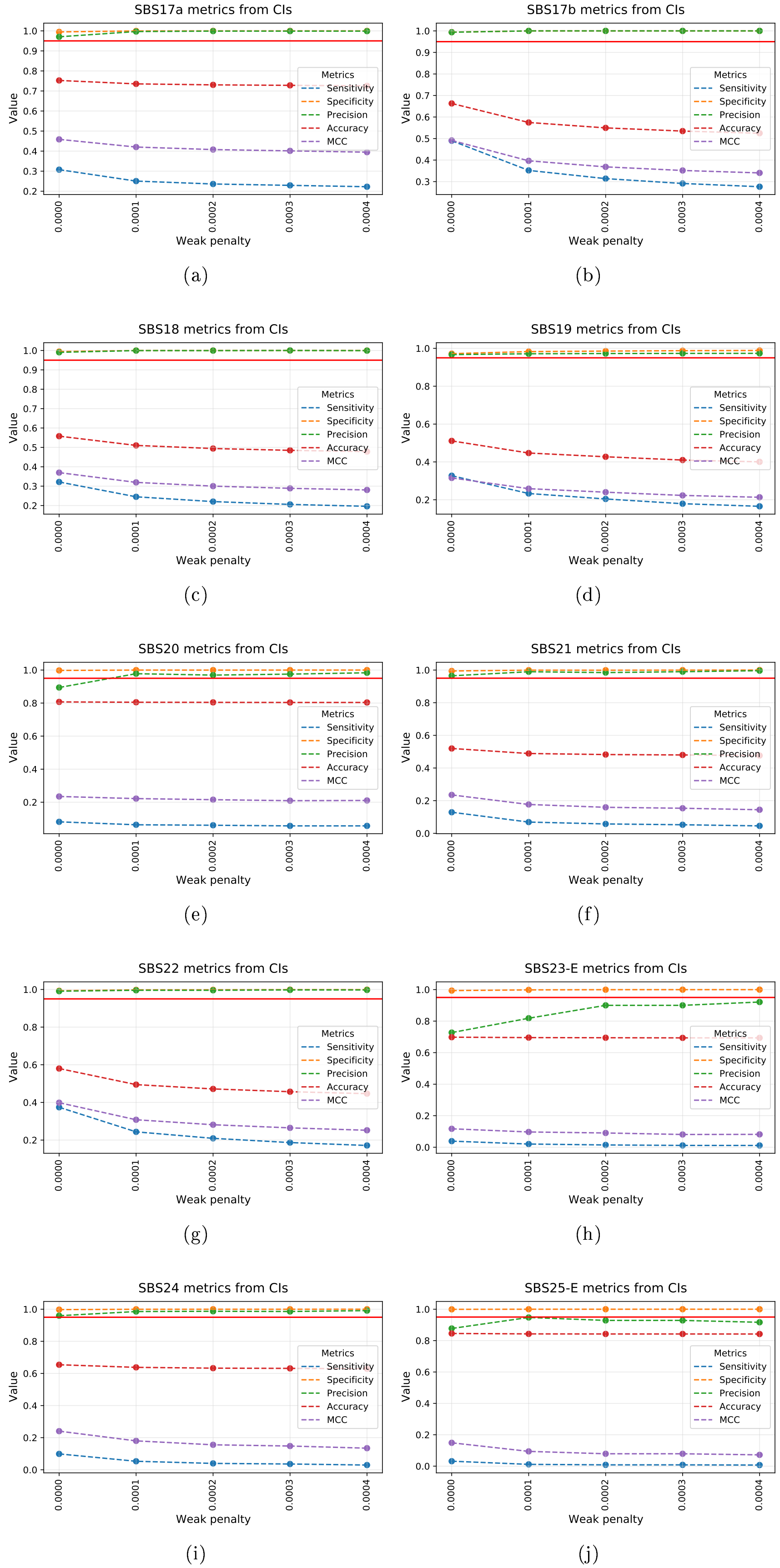


**Figure 4 MSA performance metrics: sensitivity, specificity, precision, accuracy and MCC (Matthews Correlation coefficient) measured for SBS signatures 17a - 25-E.**

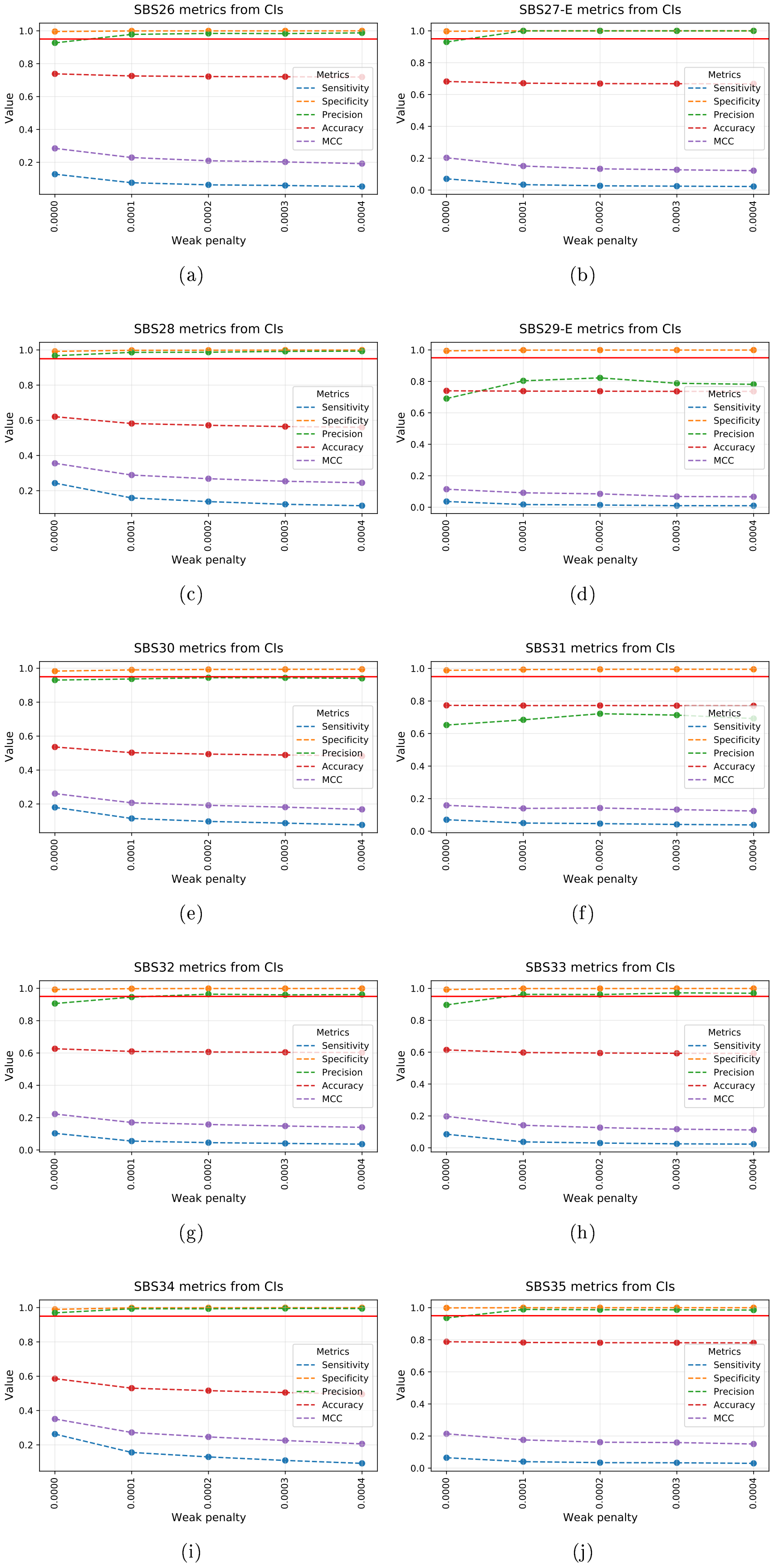


**Figure 5 MSA performance metrics: sensitivity, specificity, precision, accuracy and MCC (Matthews Correlation coefficient) measured for SBS signatures 26-35.**

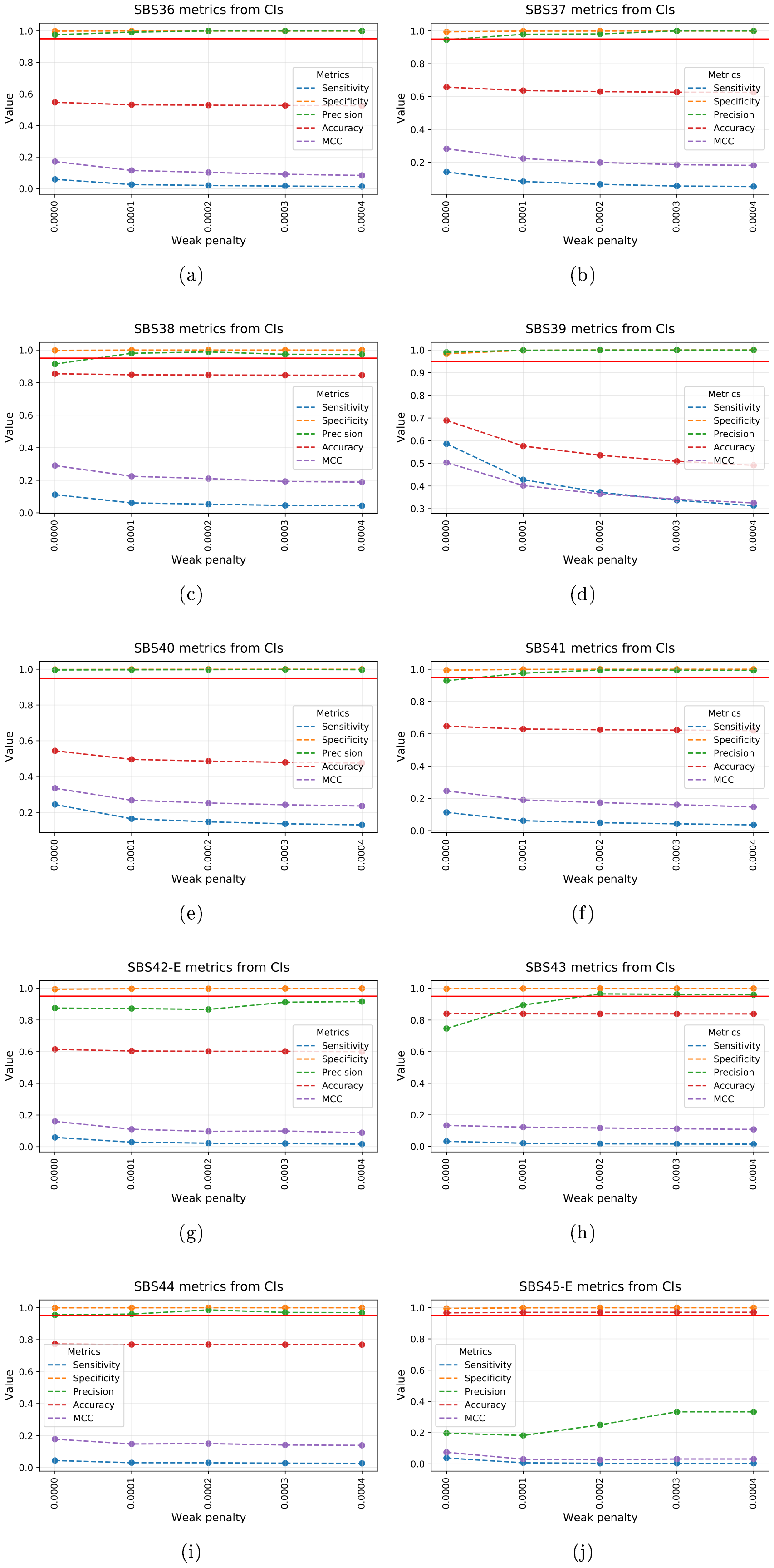


**Figure 6 MSA performance metrics: sensitivity, specificity, precision, accuracy and MCC (Matthews Correlation coefficient) measured for SBS signatures 36- 45-E.**

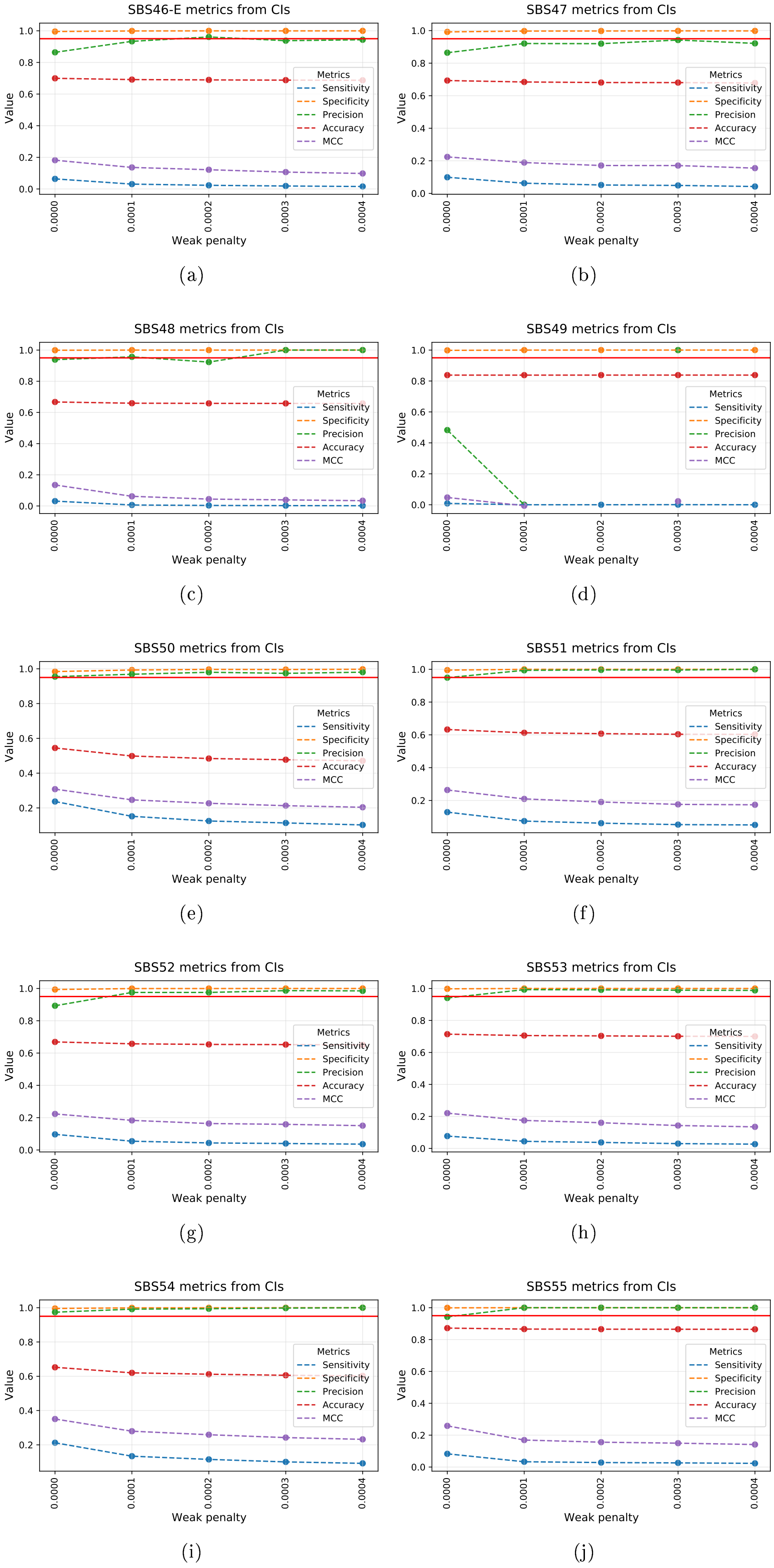


**Figure 7** MSA performance metrics: sensitivity, specificity, precision, accuracy and MCC (Matthews Correlation coefficient) measured for SBS signatures 46-E -55.

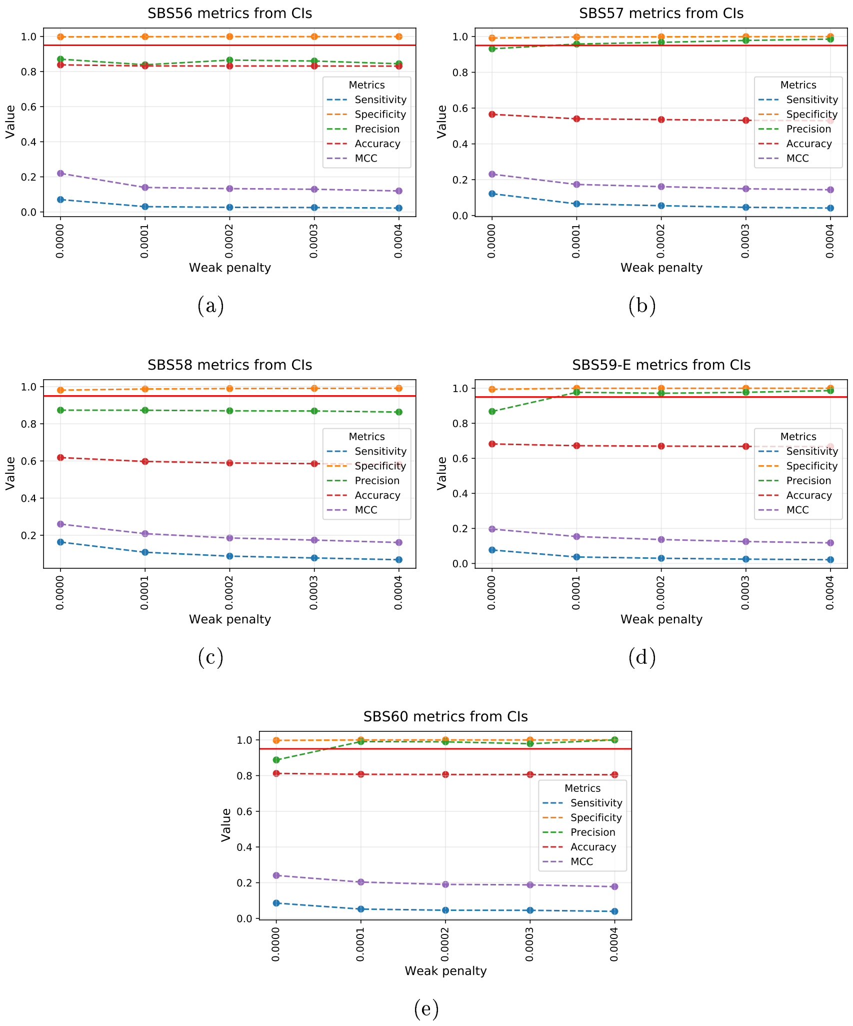


**Figure 8 MSA performance metrics: sensitivity, specificity, precision, accuracy and MCC (Matthews Correlation coefficient) measured for SBS signatures 56-60.**

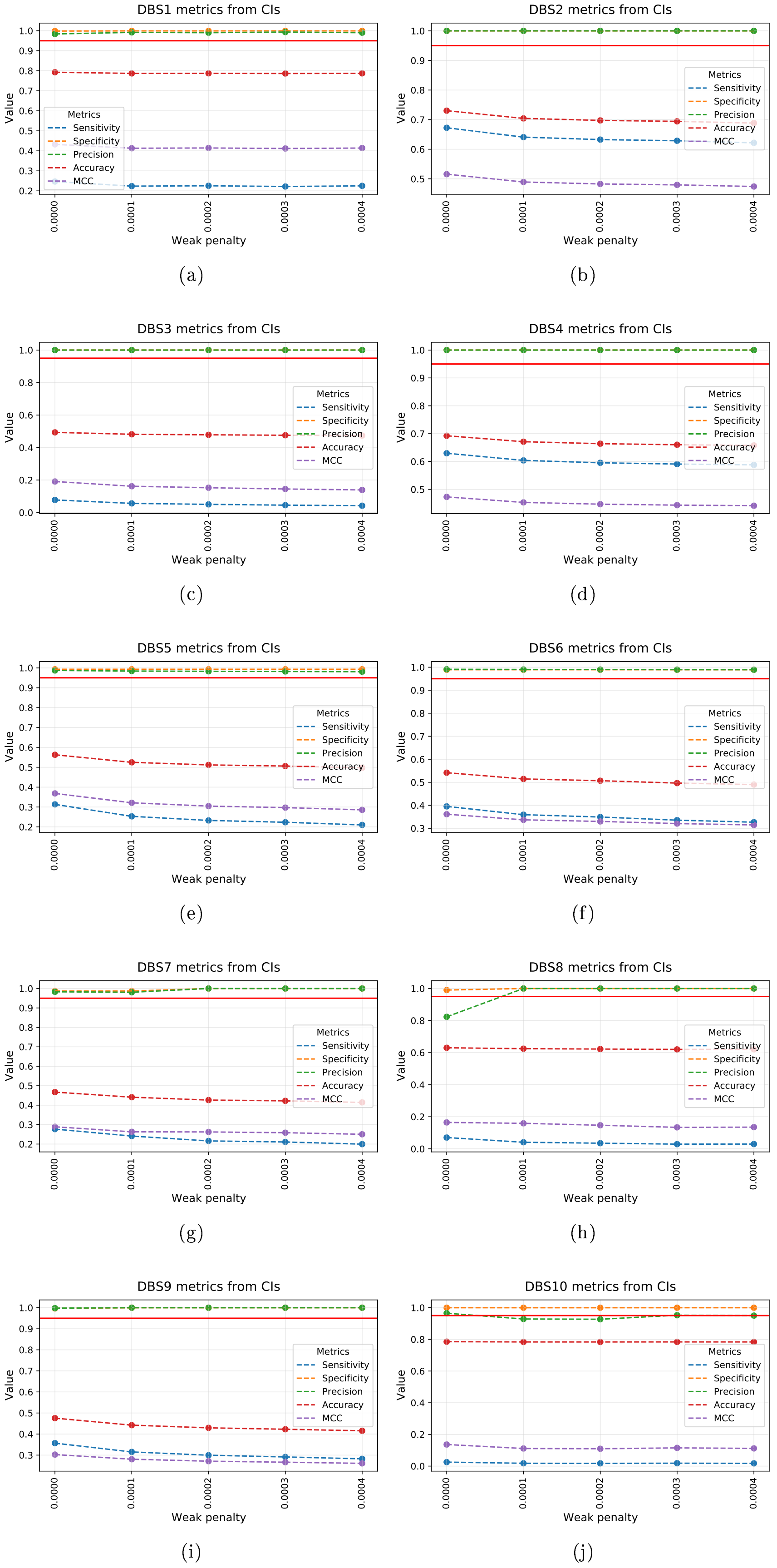


**Figure 9** **MSA performance metrics: sensitivity, specificity, precision, accuracy and MCC (Matthews Correlation coefficient) measured for DBS signatures 1-10.**

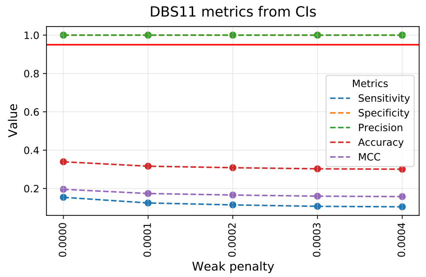


(a)

**Figure 10 MSA performance metrics: sensitivity, specificity, precision, accuracy and MCC (Matthews Correlation coefficient) measured for DBS signature 11.**

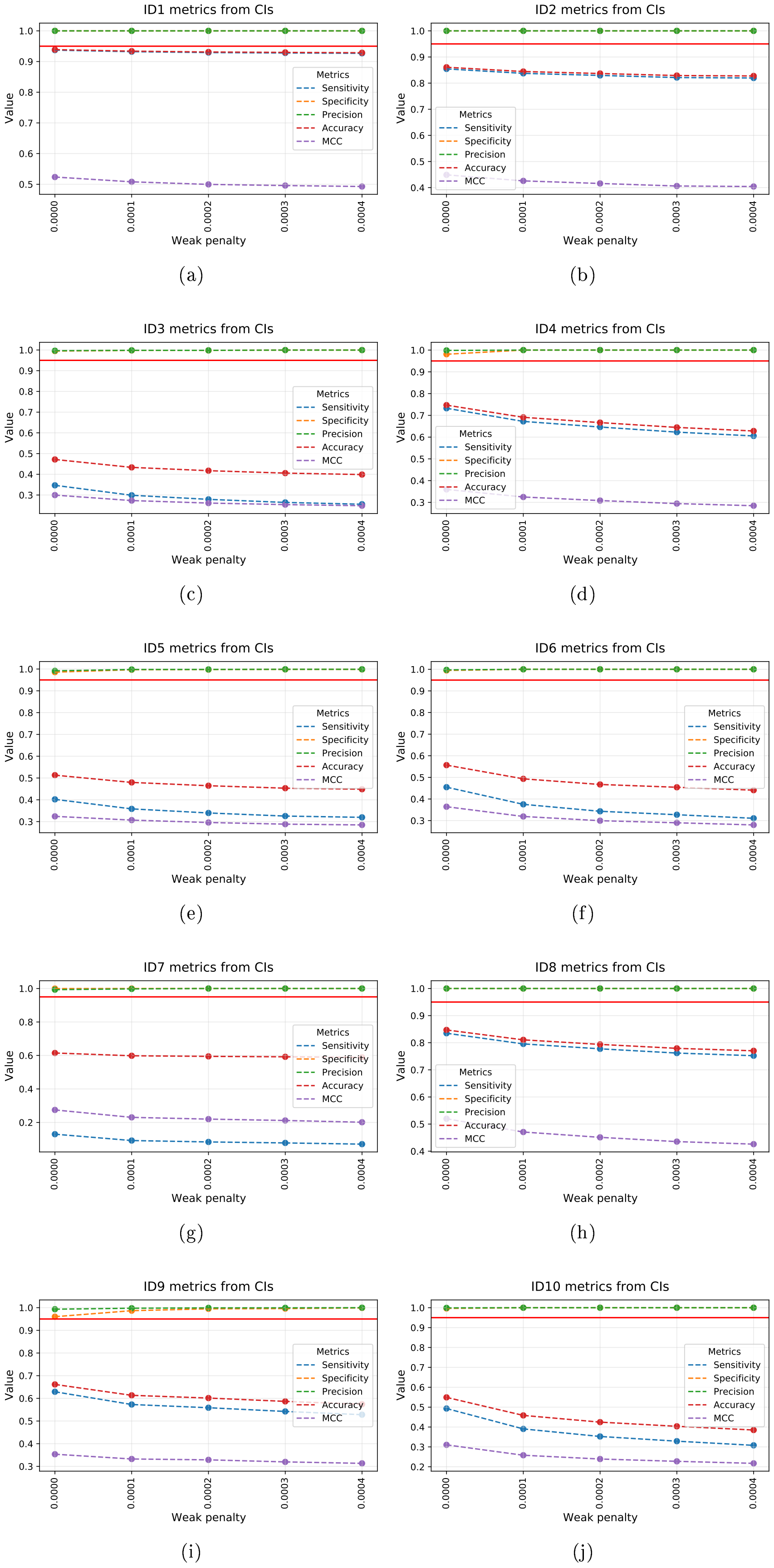


**Figure 11** MSA performance metrics: sensitivity, specificity, precision, accuracy and MCC (Matthews Correlation coefficient) measured across ID signatures 1-10.

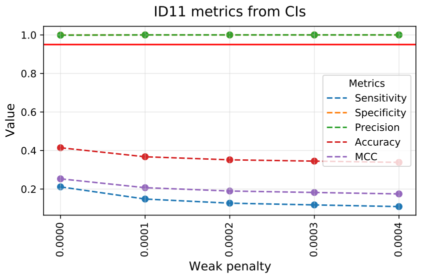


(a)

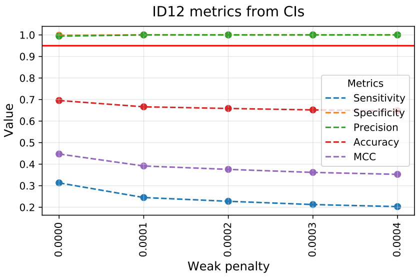


(b)

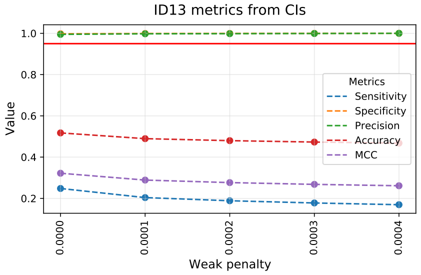


(c)

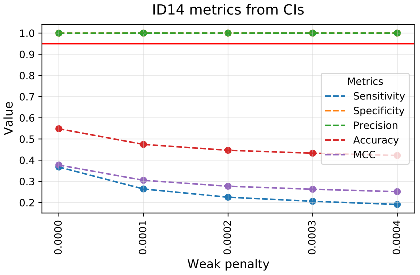


(d)

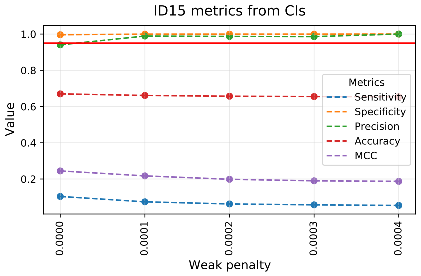


(e)

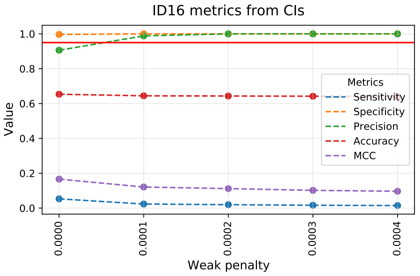


(f)

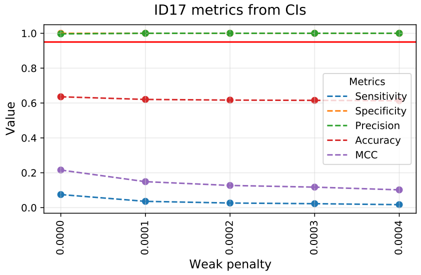


(g)

**Figure 12** MSA performance metrics: sensitivity, specificity, precision, accuracy and MCC (Matthews Correlation coefficient) measured across ID signatures 11-17.
